# Frequency of Sexual Dysfunction in Patients Treated with Desvenlafaxine: A Prospective Naturalistic Study

**DOI:** 10.3390/jcm8050719

**Published:** 2019-05-21

**Authors:** Angel L. Montejo, Joemir Becker, Gloria Bueno, Raquel Fernández-Ovejero, María T. Gallego, Nerea González, Adrián Juanes, Laura Montejo, Antonio Pérez-Urdániz, Nieves Prieto, José L. Villegas

**Affiliations:** 1 Nursing School E.U.E.F., University of Salamanca, 37004 Salamanca, Spain; 2 Instituto de Investigación Biomédica de Salamanca, Servicio de Psiquiatría, Hospital Universitario de Salamanca, 37007 IBSAL, Spain; jbecker@saludcastillayleon.es (J.B.); mtgallego@saludcastillayleon.es (M.T.G.); perurdan@ono.com (A.P.-U.); nprietom@saludcastillayleon.es (N.P.); jlvillegas@saludcastillayleon.es (J.L.V.); 3 Departamento de Psiquiatría, Universidad de Salamanca, 37004 Salamanca, Spain; gloriabueno@usal.es; 4 Servicio Navarro de Salud, 31008 Osasunbidea, Spain; rqfo25@gmail.com; 5 Departamento de Estadística, Universidad de Salamanca, 37004 Salamanca, Spain; nerea_gonzalez_garcia@usal.es; 6 Atención Primaria de Salamanca, 37900 SACYL, Spain; ajuanesd@yahoo.es; 7 Barcelona Bipolar and Depressive Disorders Program, Institute of Neurosciences, University of Barcelona, IDIBAPS, CIBERSAM, Hospital Clinic of Barcelona, 08401 Catalonia, Spain; laumonteg@gmail.com

**Keywords:** desvenlafaxine, sexual dysfunction, antidepressant, treatment, prsexdq-salsex questionnaire, switching strategy

## Abstract

Despite being clinically underestimated, sexual dysfunction (SD) is one of the most frequent and lasting adverse effects associated with antidepressants. Desvenlafaxine is an antidepressant (AD) with noradrenergic and serotonergic action that can cause a lower SD than other serotonergic ADs although there are still few studies on this subject. Objective: To check the frequency of SD in two groups of depressive patients: one group was desvenlafaxine-naïve; the other was made up of patients switched to desvenlafaxine from another AD due to iatrogenic sexual dysfunction. A naturalistic, multicenter, and prospective study of patients receiving desvenlafaxine (50–100 mg/day) was carried out on 72 patients who met the inclusion criteria (>18 years old and sexually active), who had received desvenlafaxine for the first time (*n* = 27) or had switched to desvenlafaxine due to SD with another AD (*n* = 45). Patients with previous SD, receiving either drugs or presenting a concomitant pathology that interfered with their sexual life and/or patients who abused alcohol and/or drugs were excluded. We used the validated Psychotropic-Related Sexual Dysfunction Questionnaire (PRSexDQ-SALSEX) to measure AD-related sexual dysfunction and the Clinical Global Impression Scale for psychiatric disease (CGI-S) and for sexual dysfunction (CGI-SD) at two points in time: baseline and three months after the commencement of desvenlafaxine treatment. Results: In desvenlafaxine-naïve patients, 59.2% of the sample showed moderate/severe sexual dysfunction at baseline, which was reduced to 44% at follow-up. The PSexDQ-SALSEX questionnaire total score showed a significant improvement in sexual desire and sexual arousal without changes in orgasmic function at follow-up (*p* < 0.01). In the group switched to desvenlafaxine, the frequency of moderate/severe SD at baseline (93.3%) was reduced to 75.6% at follow-up visit. Additionally, SD significantly improved in three out of four items of the SALSEX: low desire, delayed orgasm, and anorgasmia at follow-up (*p* < 0.01), but there was no significant improvement in arousal difficulties. The frequency of severe SD was reduced from 73% at baseline to 35% at follow-up. The CGI for psychiatric disease and for sexual dysfunction improved significantly in both groups (*p* < 0.01). There was a poor tolerability with risk of treatment noncompliance in 26.7% of patients with sexual dysfunction due to another AD, this significantly reduced to 11.1% in those who switched to desvenlafaxine (*p* = 0.004). Conclusion: Sexual dysfunction improved significantly in depressed patients who initiated treatment with desvenlafaxine and in those who switched from another AD to desvenlafaxine, despite this, desvenlafaxine treatment is not completely devoid of sexual adverse effects. This switching strategy could be highly relevant in clinical practice due to the significant improvement in moderate/severe and poorly tolerated SD, while maintaining the AD efficacy.

## 1. Introduction

Sexual dysfunction (SD) is one of the most frequent and lasting adverse effects caused by serotonergic antidepressants (ADs). However, unfortunately, it is underestimated in the data elaborated by pharmaceutical companies in post-registration studies, where frequencies of 2–16% are reflected [1,2], much lower than those found in specific case series studies [3,4,5], systematic reviews [6,7], and meta-analyses [8]. The reasons for this underestimation could be that the frequency of SD is obtained from clinical trials designed to find efficacy; these are usually unreliable short-term studies because they can include either sexually inactive patients or lack specific sexual dysfunction questionnaires, counting only spontaneous communications of sexual adverse events.

The real prevalence of SD secondary to treatment with ADs calculated in clinical practice is much higher, as has been demonstrated in studies including long series of patients [9,10]. Using specific questionnaires such as the Psychotropic-Related Sexual Dysfunction Questionnaire (PRSexDQ-SALSEX) [11,12] or the Changes in Sexual Function Questionnaire (CSFQ) [13], a prevalence of SD secondary to Selective Serotonin Reuptake Inhibitors (SSRI) of between 50–79% in sexually active patients has been shown [14,15,16,17], increasing to more than 80% in healthy volunteers who received SSRI for at least eight weeks [18,19].

In a recent study using the PRSexDQ-SALSEX to evaluate the frequency and tolerance of sexual dysfunction in the majority of ADs approved for the treatment of depression, such as SSRI, serotonin-noradrenaline reuptake inhibitors (SNRIs), clomipramine, agomelatine, bupropion, and mirtazapine, 79% patients showed sexual dysfunction, as indicated by a total score ≥3 on the PRSexDQ-SALSEX; 64% showed moderate-severe sexual dysfunction, with no differences between men and women on these outcomes [5]. Sexual dysfunction is extremely common in patients receiving ADs, especially serotonergic ones, with a significantly lower frequency of SD associated with non-serotonergic ADs such as mirtazapine, agomelatine, and bupropion. The consequences of this adverse side effect range from the deterioration of the quality of life to quitting treatment due to the sexual dysfunction which occurs in 20–35% of patients [15,20], with the consequent risk of relapse and other negative repercussions related to depression [21,22].

The most common symptoms of SD are a decrease in desire and a delay in achieving orgasm. Erectile dysfunction is less common, although paroxetine, citalopram, and venlafaxine produce it in about 30–40% of patients at usual therapeutic doses [1,5,13,20]. Anorgasmia or lack of ejaculation is the worst side effect tolerated by patients [15]. Unfortunately, SDs are rarely examined routinely by physicians and only 15–40% of patients report it spontaneously, despite the high frequency observed in those treated with AD [1,5,13,15] and with antipsychotics [23,24]. Therefore, it is necessary to use specific questionnaires to detect SDs and its influence on the quality of life of the patient and his/her partner in the short, medium, and long term. This feature of sexual tolerability should be considered carefully in the selection of an AD [25].

Other ADs, with non-serotonergic mechanisms of action (agomelatine, mirtazapine, bupropion) have been shown to produce little deterioration in the sexuality of patients in controlled studies compared to serotonergic ADs in medium- and long-term case series [26,27,28,29]. However, it might be possible that the clinical effect of non-serotonergic compounds was insufficient in some groups of patients in need for a serotonergic effect to deal with symptoms such as obsessive-compulsive behavior or in severely depressed patients.

Dual SNRIs, mixed inhibitors of the reuptake of serotonin and noradrenaline (duloxetine and desvenlafaxine), may have a lower frequency of SD, although there are contradictory data regarding this in several studies with varying designs [15,30]. The evidence for mirtazapine having an advantage over selective serotonin reuptake inhibitors (SSRIs) is lacking and there are currently not sufficient data as regards the effects of desvenlafaxine. Usually, sexual function data come from clinical trials on efficacy which include moderately depressed patients and do not select the trial population according to inclusion and exclusion criteria in terms of prior activity and sexual satisfaction.

Well-designed comparative studies of present ADs with a direct assessment of sexual side effects as the primary outcome measure are scarce [31]. On the other hand, ADs can improve sexual function in depressed patients. There is less frequency of SD in patients who respond better to treatment compared to those who do not significantly improve in the medium and long term [15]. With respect to this, the effect of duloxetine on the patient’s sexual life seems to be linked to the AD effect. SD is more frequent in populations that show a lower response to ADs compared to those that respond adequately [32]. On the other hand, vortioxetine, with a serotonergic, noradrenergic, and dopaminergic multimodal mechanism of action, seems to be associated with lower SD according to the data derived from clinical registration trials [33,34] and a specific study of switching to vortioxetine due to sexual dysfunction [35]; however, additional data obtained with naturalistic designs in real clinical practice are needed to corroborate this.

The SD managing approach includes dose reduction, the addition of an antidote, such as an inhibitor of phosphodiesterase type 5 (IPD5) if there is erectile dysfunction, AD withdrawal for 24–48 h before intercourse in the case of anorgasmia, and a change of treatment to another non-serotonergic AD [5,36,37,38]. None of these methods are free from risk, which can include relapses or the appearance of new adverse side effects concomitant to the change of treatment. Therefore, it is necessary to adequately clarify the role that different ADs play in the appearance of SD and the deterioration of the quality of life that they can produce in the patient. To start with, drugs that do not have deteriorating effects on sexual life in sexually active patients should always be taken into account in patients with depression. On the other hand, given that SD is more frequent with some ADs, it should always be considered in the investigation of new molecules, seeking better tolerability profiles in the medium and long term [39].

There are still few studies that specifically analyze the influence of desvenlafaxine on the sexual function of patients. The risk of SD associated with desvenlafaxine treatment has been studied in a post-hoc analysis of a clinical trial compared to placebo using the ASEX. After 12 weeks, orgasm delay was observed at a higher rate than placebo in men, but not in women. Other sexual functions such as desire and arousal were not affected [40]. In another randomized double-blind study, no differences were found between desvenlafaxine (50–100 mg) and placebo [41]. The data suggest that there could be higher SD with 100 mg/day, but these data were not conclusive since no statistically significant differences were obtained. After an integrated analysis of short-term, randomized, double-blind, placebo-controlled registration studies for major depressive disorder (50–400 mg/day) with desvenlafaxine vs. placebo for eight weeks, very few adverse sexual effects were found with desvenlafaxine: erectile dysfunction in men (7% vs. 1% with placebo) and anorgasmia in women (1% vs. 0%) [42]. This low frequency of SD data does not seem to be in line with what was expected of a drug with a serotonergic mechanism of action. This could be due to the fact that these studies were not specifically designed to find differences in sexual function with samples properly selected using inclusion and exclusion criteria, or because only spontaneous communications were taken into account over a short period of eight weeks.

Until now, there have been hardly any naturalistic studies specifically designed to evaluate the SD associated with desvenlafaxine under the conditions of usual clinical practice. The methodology, to obtain adequate results, should include patients with a previous active sexual life and lack of another pathology or concomitant medication that might affect sexual function, in order to determine the causality that the drug (and not other associated factors) plays in the possible dysfunction of sexual life.

In a recent study with a naturalistic design in patients with depression or anxiety disorder treated with fluoxetine, mirtazapine, escitalopram, sertraline, and desvenlafaxine, the sexual function of 209 patients was evaluated using the PRSexDQ-SALSEX questionnaire at baseline and at six weeks. Twenty-one percent showed sexual dysfunction at the beginning of treatment and this increased to 41% in week 6. With regard to individual questionnaire items, by week 6, sexual desire improved, but erectile and ejaculatory function in men and orgasmic function in women worsened. Fluoxetine and sertraline were associated with impaired sexual function, whereas mirtazapine was associated with favorable sexual function. At week 2, mirtazapine and desvenlafaxine were predictors of favorable sexual outcome. Additionally, sexual dysfunction was more frequent in men than in women [43].

The main goal of this study is to evaluate the frequency of SD in patients treated with desvenlafaxine under usual clinical practice conditions using two independent groups: the first one with new patients, desvenlafaxine-naïve, defined as patients who never before received an AD treatment, and the second group with patients who were changed to desvenlafaxine due to experiencing SD secondary to another AD.

## 2. Experimental Section

A naturalistic, multicenter, prospective study was conducted in patients treated with desvenlafaxine (at a dose as recommended by the data sheet and usual clinical practice of 50–100 mg/day) or another AD. We included 72 patients who met the inclusion and exclusion criteria into two groups: group A, desvenlafaxine-naïve, with 27 patients receiving desvenlafaxine for the first time, and group B, with 45 patients switched to desvenlafaxine for presenting SD caused by another AD.

### 2.1. Study Population

Inclusion and exclusion criteria were used to guarantee the validity of the sample, avoiding possible confounding factors. Patients were consecutively included if they fulfilled the following inclusion criteria for group A (desvenlafaxine-naïve): over 18 years old, sexually active (defined as at least one sexual activity in the last six months), who started treatment with desvenlafaxine 50–100 mg/day according to usual clinical practice. The following inclusion criteria were used for group B (switched to desvenlafaxine): over 18 years old, receiving treatment with AD for at least eight weeks prior, a history of self-reported normal sexual functioning before the prescription of the AD, excluding a mildly impaired libido (libido impairment is part of the depressive symptoms). Exclusion criteria: patient receiving more than one AD or requiring concomitant treatment that may influence sexual activity (antipsychotics, antihypertensives, beta-blockers, sex hormones, opiates), alcohol and/or drug abuse and serious medical illness that could cause sexual dysfunction. Patients were allowed to receive treatment with benzodiazepines. In the baseline visit, information on patient demographics, psychiatric history, and the treatments that the patient was receiving were recorded.

### 2.2. Measurement Scales

Sexual function was evaluated with the Psychotropic-Related Sexual Dysfunction Questionnaire (PRSexDQ-SALSEX), which has shown good psychometric properties both in patients with depression [9] and in patients with schizophrenia [10]. In patients with depression, the PRSexDQ-SALSEX has shown adequate internal consistency, with a Cronbach’s alpha value of 0.93, and adequate construct validity [9]. As may be expected, the PRSexDQ-SALSEX showed a high correlation with the Clinical Global Impression Scale for sexual dysfunction (*r* = 0.79) and a moderate correlation with Hamilton Depression Rating Scale scores (*r* = 0.63). The PRSexDQ-SALSEX also showed good discrimination between naïve and pretreated depressed or dysthymic patients, with statistically significant differences between those groups of patients. In brief, the PRSexDQ-SALSEX has seven questions, and is hetero-applied by the evaluator. The first two questions use a yes/no format to record whether patients have noticed any change in their sexual function since they initiated treatment and whether the sexual dysfunction was spontaneously reported. The next four questions (items 1 to 4) employ a four-point scale, from no problem to severe problem, to assess the presence and severity of decreased libido, delayed ejaculation/orgasm, lack of ejaculation/orgasm, and difficulties with having or maintaining erection/lubrication. The last question (item 5) evaluates the tolerability of the changes in sexual functioning on a four point scale: 0, No sexual dysfunction; 1, Well, no problem due to this reason; 2, Fair, the dysfunction bothers him or her, although he or she has not considered discontinuing the treatment for this reason, or it interferes with the couple’s relationship; 3, Poor, the dysfunction presents an important problem, and he or she has considered discontinuing treatment because of it, or it seriously interferes with the couple’s relationship. These five latter items (i.e., items 1 to 5) account for the total score of the PRSexDQ-SALSEX, which ranges from 0 to 15. According to this total score, patients may be categorized as having no sexual dysfunction (a score of 0 or the item 1 (libido) scoring 1 and the item 5 (tolerability) scoring 1), or having mild (total score of 3–5) dysfunction, provided that no item scores ≥2 (i.e., provided that the patient does not have moderate sexual dysfunction in a specific dimension), moderate (total score of 6–10 or an item scoring 2, provided that no item scores 3 (i.e., provided that the patient does not have severe sexual dysfunction in a specific dimension)) or severe (total score of 11–15 or an item scoring 3) sexual dysfunction.

The severity of the psychiatric disorder was evaluated at baseline with the 7-point Clinical Global Impression of Severity Scale (CGI) [44], as well as the CGI of Sexual Dysfunction (CGI-SD), which is a clinician-rated instrument identical to the CGI used for the assessment of psychopathology. Additionally, a 7-point CGI of Change scale was used for psychiatric severity (CGI-CS) [44] and for sexual dysfunction (CGI-CSD) in the follow-up visit, three months after the baseline visit. The research team consisted of eight researchers previously trained in the application of SALSEX belonging to the Department of psychiatry of the University Hospital of Salamanca (seven psychiatrists) and the Primary Care Service of Salamanca (one general practitioner) between June 2015 and June 2016.

### 2.3. Ethical Aspects

The protocol was favorably evaluated by the Research Ethics Committee of Salamanca (CEIC) in November 2014. It was classified by the Spanish Agency for Medicines and Healthcare Products (AEMPS) as a post-authorization study with prospective follow-up (EPA-SP study) and was authorized later by the General Directorate of Public Health of Castilla y León, dated February 2015, with the code AMG-DES-2014-01. All patients signed an informed consent following the international norms and procedures of medical research in humans using the declaration of Helsinki of the World Medical Association of 1964.

### 2.4. Sample Size

There are no specific published data so far to estimate the sample size based on the effect size. There is only a post-hoc analysis of a pivotal clinical trial in which desvenlafaxine did not show any significant difference vs. placebo in the mean change of ASEX scale at endpoint (12 weeks of treatment), with a mean reduction in ASEX of −1.13 (SD = 0.47) in the males group and −1.93 (SD = 0.37) in the females group treated with desvenlafaxine [40]. This is not a study primarily designed to evaluate sexual functioning and its design presents relevant differences to the design of our study, so the values needed to calculate sample size are not available. We have therefore considered this as a pilot study to evaluate the changes in sexual functioning assessed with the PRSexDQ-SALSEX questionnaire, with mean change global score as the main outcome, estimating a sample size of 50 subjects (25 per group) to detect differences in sexual functioning after three months of treatment with desvenlafaxine. A study withdrawal rate of 15% was assumed.

### 2.5. Statistical Analysis

Valid data were analyzed from the database which was created ad-hoc. The main variable “mean change in PRSexDQ-SALSEX global score from baseline to follow-up visit” and secondary variables “mean change in PRSexDQ-SALSEX global score at follow-up visit”, “mean change in each item of PRSexDQ-SALSEX score at follow-up visit”, and “mean change in all CGIs” were analyzed. The distribution of the quantitative variables was examined using the Kolmogorov–Smirnov test. Later, they were described using mean and standard deviation (mean ± standard deviation (sd)) in the case of data that followed the normal distribution, otherwise, median and interquartile range (the first quartile subtracted from the third quartile) (median ± IQR) were used. The differences between independent groups of quantitative variables were evaluated using the Student’s t-test for data of normal distribution, or otherwise using the non-parametric Mann–Whitney U test. The differences in the response to quantitative variables between paired groups were examined using the Student’s t-test for paired data (for normally distributed variables) or the Wilcoxon test (for non-parametric data). The descriptive analysis of categorical qualitative variables was carried out through frequencies and percentages. Chi-square test and Fisher’s exact test were used to analyze the association of qualitative variables. The statistical package SPSS, version 22.0, (IBM Corporation, Armonk, NY, USA) was used for the statistical analysis, with strict quality control procedures. The results were considered significant if *p* < 0.05.

## 3. Results

### 3.1. Study Population

Initially, 109 patients were included, of whom 37 did not attend the follow-up visit (nine in group A and 28 in group B, 10 men and 27 women), so they were lost for the study. Finally, 72 patients were included (52 women and 20 men) with a mean age of 43.4 ± 11.8 diagnosed with affective pathology (major depression, single episode, 47.2%, recurrent major depression, 12.5%, anxious depressive disorder, 8.3%, dysthymia, 13.9%, adaptive disorder, 8.3% and others, 9.8%). They were diagnosed following the International Classification of Diseases 10th Revision (ICD-10) criteria [45]. Of the total of patients who completed the study, 37.5% were from group A (desvenlafaxine-naïve) and the remaining 62.5% were from group B (changed to desvenlafaxine from a different AD). Group A consisted of 27 patients (22 women and 5 men, average age 42.7 ± 10.9) and group B of 45 patients (30 women and 15 men, average age 43.87 ± 12.34). The patients switched to desvenlafaxine coming from other previous treatments consisted of 16 patients with escitalopram (36%), 10 duloxetine (22%); 6 venlafaxine (13%); 5 sertraline (11%); 4 paroxetine (9%); 4 Fluoxetine (9%). In 52.3% of the cases, a gradual change was made, and in the rest of the cases, a sudden change was made to clinical criteria without using a washout period.

### 3.2. SALSEX Questionnaire

The spontaneous communication of SD (item B of SALSEX) occurred in 27.3% of patients in group A and in 35% of group B. The overall SALSEX results for group A and B are shown in Table 1.

**Figure 1:**
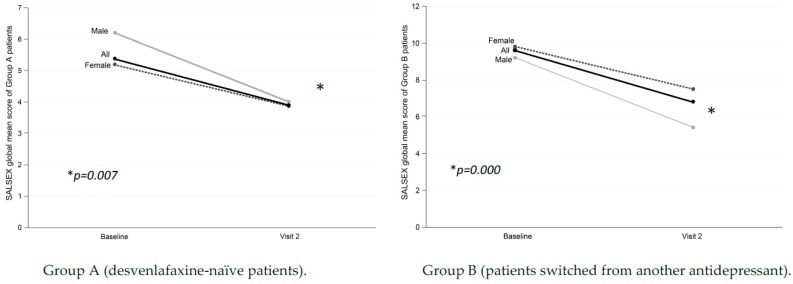
Differences in overall sexual function from baseline at three months in patients treated with desvenlafaxine.

In group A, an overall global SALSEX score was obtained at the baseline visit of 5.4 ± 4.3 (with 0 = no SD and 15 = maximum dysfunction), indicating a degree of mild/moderate initial SD in the sample of desvenlafaxine-naïve patients. The overall score on the SALSEX scale between the baseline (5.4 ± 4.3) and follow-up visit (3.9 ± 3.6) was reduced in a highly significant way (*p* = 0.007) (Figure 1), showing a clinical improvement in the sexual function of patients who started treatment with desvenlafaxine at follow-up. In the desvenlafaxine-naïve group, the intensity of the SD was reduced by 28%.

Patients in group B (changed to desvenlafaxine) obtained a global average score on the SALSEX scale at the baseline visit of 9.6 ± 3.6 and 6.8 ± 3.9 in the follow-up visit, resulting in this highly significant difference (*p* = 0.000) (Figure 1). The improvement in overall sexual function measured with total SALSEX was greater in group B (all patients suffered from SD secondary to previous treatment) as their initial SD was reduced by 36% in the second visit.

**Figure 2:**
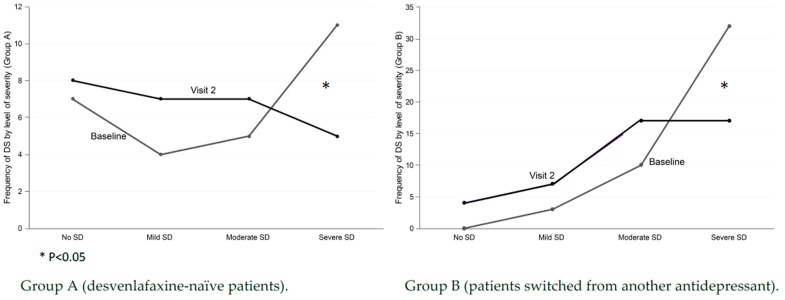
Differences in sexual level of severity from baseline at three months in patients treated with desvenlafaxine.

Taking into account the total score of the SALSEX, the severity of the SD was distributed into several groups: mild (Salsex = 1–5 points); moderate (Salsex = 6–10 or any item = 2); and intense (Salsex = 11–15 or any item = 3). Desvenlafaxine-naïve patients showed a significant decrease in severe SD at follow-up (11 patients in baseline vs. 5 patients in the follow-up visit) (*p* < 0.05) (Figure 2). In group B of patients changed to desvenlafaxine, there was a decrease in the group of intense SD from the baseline visit (32 patients in the baseline vs. 17 in the follow-up visit) (*p* < 0.05).

The overall frequency of baseline SD (defined as SALSEX score ≥3) was 74% of 27 patients in group A desvenlafaxine-naïve (14.8% mild, 18.5% moderate, and 40.7% intense) and 70.3% at follow-up (mild 25.9%, moderate 25.9%, and intense 18.5%). In desvenlafaxine-naïve patients, 59.2% of the sample showed clinically relevant (moderate/severe) sexual dysfunction at baseline that was reduced to 44% at follow-up. A similar global frequency was observed in SD from the beginning but with a significant decrease in intensity (*p* = 0.045), reducing the percentage of cases with severe SD. In group B, the overall frequency of SD was 100% at baseline (6.7% mild, 22.2% moderate, and 71.1% intense), decreasing to 91.2% at follow-up (15.6% mild, 37.8% moderate, and 37.8% intense). In this group, the frequency of clinically relevant (moderate/severe) SD at baseline (93.3%) was reduced to 75.6% at follow-up. A significant reduction in the intensity of SD after switching to desvenlafaxine was observed (*p* = 0.011).

#### 3.2.1. Gender Differences

**Figure 3:**
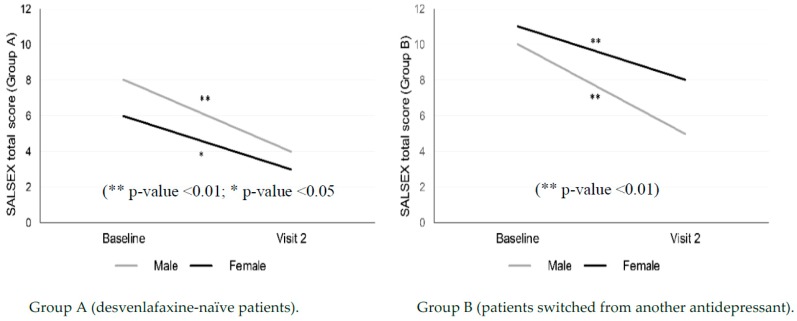
Gender differences in overall sexual function from baseline at three months in patients treated with desvenlafaxine.

Analyzing the differences between men and women, significant improvements were found in the overall SALSEX scale in both men and women for the two treatment groups (Table 2 and Figure 3). The overall improvement was more intense in men of both groups (a 38% improvement in group A in the follow-up visit and 41.3% in group B), although it is necessary to take into account that the sample of men is much lower in the study and this can influence the results (52 females vs. 20 males).

#### 3.2.2. Differences by Dose

The patients in the study mostly received doses of 50 mg/day and 100 mg/day of desvenlafaxine. The mean dose of group A was 53.7 ± 13.3 mg/day at the baseline visit and 63.7 ± 24.7 at follow-up. Patients in group B received doses of 62.5 ± 22.1 mg/day of desvenlafaxine at the baseline visit and 63.44 ± 26.39 mg/day at follow-up. In group A, 92.6% (*n* = 25) of them received a dose of 50 mg/day and the remaining 7.4% (*n* = 2), a dose of 100 mg/day at the baseline visit; while in the follow-up visit 66.7% (*n* = 18) and 29.6% (*n* = 8) of them received doses of 50 and 100 mg, respectively. In the case of patients in group B, at the baseline visit, 75% of patients (*n* = 18) received a dose of 50 mg/day and the remaining 25% (*n* = 6), a dose of 100 mg/day. At follow-up, 60% of patients (*n* = 27) received a dose of 50 mg/day, while only 28.9% (*n* = 13) received a dose of 100 mg/day. The possible association between desvenlafaxine dose and PRSexDQ-SALSEX global score was examined. In this sense, there were no statistically significant differences in the overall PRSexDQ-SALSEX scale score in the baseline visit or in the follow-up visit between those who received a dose of 50 mg/day and those who received a dose of 100 mg/day in any of the two groups of patients. At the descriptive level, overall SALSEX scores were lower in those patients who received a higher dose of desvenlafaxine, although the percentage of cases that received a dose of 100 mg/day of desvenlafaxine was very small and conclusive results cannot be extracted regarding this.

#### 3.2.3. Analysis of the PRSexDQ-SALSEX Dimensions

**Figure 4:**
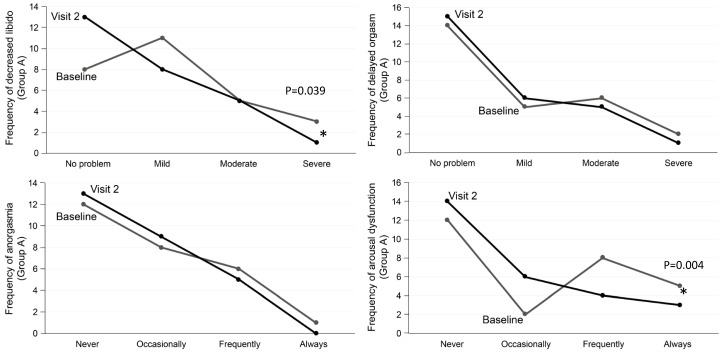
Group A. Desvenlafaxine-naïve patients. Changes in sexual functioning at follow-up.

The analysis of the individual items in the desvenlafaxine-naïve group showed that no worsening was observed in any of the items in the follow-up visit compared with the baseline visit. On the contrary, there was a significant improvement in desire (*p* = 0.039) and in sexual arousal (*p* = 0.004) without significant change in orgasm, so we can say that sexual affectation by desvenlafaxine in the studied sample is scarce (Figure 4).

**Figure 5:**
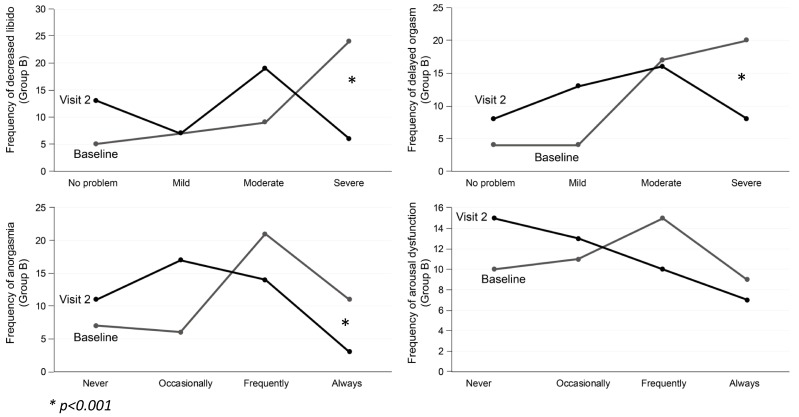
Group B. Patients switched to desvenlafaxine. Changes in sexual functioning at follow-up.

In patients that switched to desvenlafaxine, significant improvements in sexual desire, delayed orgasm, and anorgasmia appear in the follow-up visit (*p* = 0.001). Regarding the item of arousal difficulty (erection in males and vaginal lubrication in women), no significant differences were found (*p* = 0.69) (Figure 5).

#### 3.2.4. Tolerability of Sexual Dysfunction

Item 5 of the SALSEX measures the tolerability and patient acceptance of SD by means of an intensity scale in which “1 = Tolerates the SD well”, “2 = Tolerates the SD with some difficulties although has not thought of dropping out of the treatment for this reason” and “3 = Tolerates the SD poorly, with it affecting his/her relationship with partner and/or has considered dropping out of the treatment for this reason”. Regarding the patients who tolerated the SD poorly and who were at risk of noncompliance (item 5 = 3) in the two groups, an improvement in the tolerability of the SD at the baseline visit was observed compared with the follow-up visit (*p* = 0.002) (18.5% of baseline poor tolerability vs. 0% in the follow-up visit in desvenlafaxine-naïve group), significantly decreasing the risk of dropouts for this reason. Similarly, patients switched to desvenlafaxine showed poor tolerability with a risk of dropout at baseline in 26.7% of cases, which significantly reduced to 11.1% at the follow-up visit (*p* = 0.004).

### 3.3. Clinical Global Impression Scales

In the scale of severity of psychiatric pathology (CGI), significant improvements were observed in both groups (group A: *p* = 0.000, group B: *p* = 0.003). Therefore, it can be considered that patients treated with desvenlafaxine showed a significant improvement in clinical situation, both when used as a starting treatment or when switching from another AD. The CGI scale of SD intensity (CGI-SD), coinciding with the results of PRSexDQ-SALSEX, showed no changes in the desvenlafaxine-naïve group but a significant improvement in the intensity of SD in patients switched to desvenlafaxine due to SD (*p* = 0.000); therefore, it can be deduced that the improvement in SD is also accompanied by improvements in the clinical situation (Table 3).

## 4. Discussion

The results of this naturalistic and prospective study show that sexual functioning improved in both patient groups (desvelafaxine-naïve patients and those switched to desvenlafaxine from another AD) measured by the overall score of the SALSEX scale. Contrary to what usually occurs in patients treated with serotonergic drugs who present a high frequency of SD, treatment with desvenlafaxine, which has a dual serotonergic and noradrenergic mechanism of action, was associated with a moderate/severe deterioration (clinically significant) in sexual functioning in 44.4% of sexually active patients who started treatment with desvenlafaxine. It is interesting that in contrast to the figures obtained in a recent study carried out with the same methodology using the PRSexDQ-SALSEX, the frequency of moderate/severe SD was higher (66% with SSRI and 75% with SNRIs (venlafaxine, and duloxetine)) [15]. In this study, desvenlafaxine presents a much more favorable profile associated with SD in desvenlafaxine-naïve patients (44.4%) despite having a mechanism of mixed serotonergic and noradrenergic action and so being considered in the group of dual ADs.

This lower deterioration of sexual function compared with other studies on serotonergic ADs that impair sexual function (as is widely described in the literature [13,19,21]) can be very relevant when choosing a drug with dual effects but with a lower ability to influence sexual functioning. In contrast to venlafaxine, which has shown a high frequency of sexual dysfunction in comparative studies including series of cases measured with the same PRSexDQ-SALSEX questionnaire [13,15], desvenlafaxine, the primary metabolite of venlafaxine, has a much lower frequency of sexual dysfunction, which could be due to the fact that desvenlafaxine is also a relatively low potency 5-HT and NE reuptake inhibitor. The lower pharmacodynamic potency in the reuptake of serotonin compared to venlafaxine might be linked to the lower effect in the dopaminergic brake, mediated by serotonin, associated with sexual dysfunction. Recently, it has been shown that paroxetine, a potent inhibitor of serotonin reuptake, influences the mechanism of AD-related sexual dysfunction through the inhibition of tyrosine hydroxylase in dopaminergic neurons related to sexual areas such as substantia nigra, pars compacta, and the ventral tegmental area but not with agomelatine in male rats [46].

In the group of patients switched to desvenlafaxine, with previous SD secondary to treatment with another AD (SSRI or SNRI), an improvement in the frequency of moderate/severe SD was observed, going from 93.3% to 75.6%. In this group in which most had severe dysfunction (and taking into account that desvenlafaxine was not devoid of sexual dysfunction in the follow-up visit), there was a significant decrease in baseline values, indicating a clinical improvement in these patients. In addition, a significant decrease was seen in those who initially showed deterioration in desire and orgasmic dysfunction without there being an improvement in sexual arousal. On the other hand, analyzing a very important aspect from the clinical point of view, the poor tolerability for patients with previous sexual dysfunction also improved significantly, decreasing from 26% to 11% in those who tolerate the SD badly (where it significantly affects the quality of life and/or the couple’s relationship and/or the patient has thought about dropping out of the treatment).

Some studies that have focused on the relationship between compliance and SD indicate that between 20–35% of patients receiving AD present a risk of dropout [13,15,19,24]. An observational study showed that the most frequent adverse effect related to the noncompliance of treatment three months after the start of an AD was sexual dysfunction (47%), followed by weight changes, gastrointestinal discomfort, and insomnia [47]. The improvement in the risk of noncompliance with the switch to desvenlafaxine could undoubtedly have very relevant implications to the medium- and long-term results, avoiding relapses and reducing the deterioration in the quality of life.

Regarding the possible differences between men and women, in our study, significant improvements were found in the overall SALSEX scale in both men and women for the two treatment groups (*p* < 0.05), with more evident improvements in males switched to desvenlafaxine. These data coincide with previous examples with large sample sizes where a higher frequency of SD was observed in females taking AD [13,15]. There are data on the generalization of SD in the general population through extensive surveys in different countries, noting that among women between 40 and 80 years old, there are frequent problems related to desire, anorgasmia, and sexual arousal, while erectile dysfunction is the most common sexual problem among males, obviously increasing with age [48,49]. Gender differences play a fundamental role in sexual activity, with males generally showing more interest in sexual activity and staying active for longer. At least 38% of males remain interested in sexuality when above 75 years old vs. 16% in females [50]. The elderly male population is of great interest, because a group of them receiving ADs which impair sexual functioning are more likely to show a worse acceptance than younger males after the appearance of SD [1,13,15].

The spontaneous communication of SD (item B of SALSEX) was found in 27.3% of patients in group A and in 35% of group B that had previously received AD. Recent findings show the high frequency of SD after AD use (78% for men and 80% for women) [15], which surpass the figures found in the general population, contrasting with the scarcity of spontaneous communications. Nevertheless, spontaneous communication has increased over the years using the SALSEX questionnaire, from 14% in 1997 [1] to 20% in 2001 [13], reaching up to 44% in 2019 [15]. This increase in spontaneous communication is possibly due to the greater knowledge and sensitivity regarding this problem in the general population, with psychiatrists, AD prescribers, and general practitioners. One factor that undoubtedly contributes to this limited communication is related to the low frequency with which prescribers systematically interview patients about the presence of SD, this seeming to be relevant to both doctors and nurses [51,52].

In relation to possible differences between the frequency of SD with 50 and 100 mg/day, the Mann–Whitney U test revealed no statistically significant differences (*p* > 0.05) in the overall score of the SALSEX scale at follow-up in any of the groups; this is possibly due to the small number of patients taking 100 mg/day. The data from this study partially confirm those obtained in pre-registration clinical trials conducted with desvenlafaxine, which indicated a better profile of sexual tolerability compared with other serotonergic ADs [42]; however, in our sample, desvenlafaxine is not exempt from sexual dysfunction at the average doses used of 63.44 mg/day (±26.39). In one of the trials on placebo-controlled desvenlafaxine using the ASEX at 12 weeks, orgasm retardation was superior to placebo in males, but not in females, without affecting desire or sexual arousal [38]. There seems to be a difference in the sexual effects when taking into account the dose used being 50 mg similar to placebo, although the studies were not carried out considering inclusion and exclusion criteria on sexual activity and the factors that may influence it. Thus, in another clinical trial designed to measure functionality, male and female outpatients with major depression were randomly assigned 12 weeks of double-blind treatment with desvenlafaxine at 50 mg/day or placebo, sexual functioning scores, measured with the ASEX, were comparable between groups [53]. In another randomized study, no differences were found in SD with desvenlafaxine at 50 mg/day, 100 mg/day, and placebo but there were suggestions that there could be higher SD in the 100 mg group although statistical significance was not achieved [39]. The existing information on the frequency of desvenlafaxine-associated SD comes from a post-hoc analysis of three double-blind and short-term clinical trials (two months) using the self-administered ASEX Scale in outpatients with depression using doses of 50–100 mg. The results indicate that SD rates were 54%, 47%, and 49% for 50 mg/day, 100 mg/day, and placebo, respectively, with adjusted odds ratios (95% confidence interval) vs. placebo of 1,205 (0.928, 1.564) and 1,129 (0.795, 1.604), respectively [54]. These results partially coincide with the SD figures found in our study with 44% of moderate/severe SD at three months in desvenlafaxine-naïve patients; however, the frequency was much higher (76%) in patients switched to desvenlafaxine due to previous AD-related sexual dysfunction after following the course for three months.

To our knowledge, this is the first study carried out in patients switched to desvenlafaxine due to SD secondary to the use of another AD (mainly SSRIs and SRNIs) so it is not possible to compare the frequency found here with previous studies. Given the characteristics of this group containing 45 patients with previous sexual dysfunction (most of them poorly tolerated) and who met inclusion and exclusion criteria to avoid the most common confounders (sexual dysfunction prior to taking the AD, previous active and satisfactory sexual life, and the absence of concomitant treatments and medical pathologies that could affect sexual function), it is possible that there were other confounding factors that contributed to the presence of sexual dysfunction, since in 75.6% of patients, SD remained at three month follow-up visit. However, the improvement in the frequency and intensity of SD was significant after the change to desvenlafaxine, followed by an improvement of three of four SALSEX items in the follow-up visit (*p* < 0.01), such as low desire, delayed orgasm, and anorgasmia, but it was not significant in arousal difficulties. Additionally, the SD intensity decreased significantly in each of the items and the frequency of severe SD was reduced from 73% at baseline to 35% at follow-up.

The Clinical Global Impression (CGI) scores for psychiatric disease (CGI-S) and for sexual dysfunction (CGI-SD) improved significantly in both groups (*p* < 0.01) at follow-up, indicating an improvement both in the previous psychiatric pathology (mainly affective disorder) and in the subjective impression of the patient on the previous SD. The patient’s CGI scales are very relevant in naturalistic studies because they indicate the subjective perception of the patient independently of the absolute value of a scale designed to measure an adverse effect. These improvements in the CGI scales coincide with the improvement in the tolerability of SD in both groups, decreasing the risk of treatment dropouts and ameliorating the quality of life.

One of the factors that may influence the sexual improvement observed in the patients switched to desvenlafaxine could well be a subjective effect underlying the patient’s decision to participate in a study to improve sexual function and compliance with their expectations of improvement, perhaps increasing sexual frequency. However, other studies with the same methodology using the SALSEX scale have shown that the change to another serotonergic AD (paroxetine) was not followed by a significant decrease in the frequency of SD (100% at baseline versus 89% at six months), but a clear improvement was found after switching to another non-serotonergic AD (amineptine) from 100% at baseline to 55% at six months. This finding suggests that the effect of the treatment itself is more powerful than the subjective effect on the patient [55]. Another dual AD with a similar mechanism of action to desvenlafaxine such as milnacipran [56] has also been shown in a prospective randomized study to improve the initial SD in parallel with the AD effect in patients from some different cultural backgrounds, such as Brazil and Europe [57]. Duloxetine, a dual-action AD, has shown lower SD scores than SSRI (23% vs. 28%), although not significant, associated with greater clinical improvement in the CGI severity after six months [30,32].

There are few studies of patients who switch to non-serotonergic drugs. One of them with a similar naturalistic and uncontrolled methodology, designed to change from fluoxetine to bupropion due to sexual dysfunction, demonstrated an important improvement in sexual function maintaining the AD response after eight weeks of follow-up [58].

Finally, the clinical improvement in this study of the change to desvenlafaxine group is of great interest, since no dropouts or relapses were observed due to lack of AD efficacy. In a similar study of switching to amineptine vs. paroxetine, 7.5% of the amineptine group had depressive relapses during the following six months of follow-up compared with none in the paroxetine group [52]. This feature is very relevant for clinical practice because the recommendations for managing AD-related SD include a change of AD to another one with a different mechanism of action as a first option. However, the clinical efficacy and risk of noncompliance or relapse related to this have not been discussed in the literature [15]. Although possibly useful in some patients receiving serotonergic AD, a reduction in the dose is not supported by convincing results and is associated with a risk of relapse.

Our study has several limitations. We did not record information on potentially confounding factors that are considered to be risk factors for the occurrence of sexual dysfunction, such as educational background, marital status, or employment status. On the other hand, patients were informed of the objectives of the study including the evaluation of sexual functioning before participating in the study. It is, therefore, possible that patients who were more motivated to participate in the study, showed better results after switching to desvenlafaxine. Additionally, since it is a naturalistic design in real-life clinical practice, there was no control group of patients who continued with the same treatment as previously, so a comparison of both groups is not possible. Despite the fact that our study was conducted under clinical practice conditions, the exclusion of some patients limited the external validity of our results. Finally, some aspects of sexual functioning, such as subjective satisfaction and sexual pain, were not evaluated, since they are not included in the PRSexDQ-SALSEX Questionnaire, but could be taken into consideration in further studies [59]. Being a naturalistic study, patients could take benzodiazepines according to usual clinical practice and these could have some effect on sexual dysfunction [60,61]; however, the doses used were low and only lasted a short time (3–6 weeks).

## 5. Conclusions

Sexual dysfunction improved significantly in depressed patients who initiated treatment with desvenlafaxine and in those that switched from another AD to desvenlafaxine; however, it was not completely devoid of sexual adverse effects. This switching strategy could be highly relevant in clinical practice due to the significant improvement of moderate/severe and poorly tolerated sexual dysfunction accompanied by the maintenance of AD efficacy. The change to desvenlafaxine can be a useful alternative in AD switching strategies to help fight iatrogenic sexual dysfunction while maintaining the efficacy of the previous drug and without the risk of dropout or relapse associated with switching to other non-serotonergic compounds. Additional ADs that do not adversely impact sexual function are needed as well as further research into how to manage this side effect in men and women. Finally, as a result of the scarcity of studies conducted in real clinical practice on the usefulness of different switching methods to combat iatrogenic SD, additional research is needed to generate new and strong evidence on the management of this important adverse event in daily clinical practice, which influences compliance, affects medium- and long-term outcomes, and deteriorates the quality of life of the patients.

## Figures and Tables

**Table 1:** Descriptive statistics of SALSEX questionnaire of groups A and B. Baseline vs. follow-up visit.

	Group A. Desvenlafaxine-Naïve			Group B. Switched to Desvenlafaxine		
	Baseline	Follow-Up Visit	*p*-Value	Baseline	Follow-Up Visit	*p*-Value
SALSEX total	5.4 (4.3)	3.9 (3.6)	0.007 **	9.6 (3.6)	6.8 (3.9)	0.000 **
Libido Decreased	1 (2)	1 (1)	0.039 *	3 (2)	2 (2)	0.000 **
Orgasm delayed	0 (2)	0 (1)	0.429	2 (1)	2 (1)	0.001 **
Anorgasmia	1 (2)	1 (1)	0.206	2 (1.5)	1 (1.5)	0.000 **
Arousal problems	1 (2)	0 (2)	0.004 **	2 (1)	1 (2)	0.069
Tolerability of SD	1 (2)	1 (2)	0.003 **	2 (1)	1 (1)	0.002 **

** = highly significant *p*-value (<0.01); * = significant *p*-value (<0.05).

**Table 2:** Gender differences. Global SALSEX score of groups A and B, at baseline and follow-up.

	SALSEX TOTAL Score		
	Baseline Median (IR)	Follow-Up Visit Median (IR)	*p*-Value
Group A			
Male (*n* = 5)	8 (10.5)	4 (6)	0.000 **
Female (*n* = 22)	6 (8.25)	3 (7)	0.043 *
Group B			
Male (*n* = 15)	10 (8)	5 (9)	0.000 **
Female (*n* = 30)	11 (3.25)	8 (5.25)	0.001 **

** = highly significant *p*-value (<0.01); * = significant *p*-value (<0.05).

**Table 3:** Clinical Global Impression of Severity Scale (CGI) scores of groups A and B, comparing baseline and follow-up visit values.

	Group A. Desvenlafaxine-Naïve			Group B. Switched to Desvenlafaxine		
	Baseline	Follow-Up Visit	*p*-Value	Baseline	Follow-Up Visit	*p*-Value
CGI Depression	4 (1.3)	1 (1)	0.000 **	3 (1)	2 (2)	0.003 **
CGI Sexual Dysfunction	3 (3)	3 (2)	0.539	5(1)	3 (1.8)	0.000 **

** = highly significant *p*-value (<0.01).
